# Clusters of orthologous genes for 41 archaeal genomes and implications for evolutionary genomics of archaea

**DOI:** 10.1186/1745-6150-2-33

**Published:** 2007-11-27

**Authors:** Kira S Makarova, Alexander V Sorokin, Pavel S Novichkov, Yuri I Wolf, Eugene V Koonin

**Affiliations:** 1 National Center for Biotechnology Information, National Library of Medicine, National Institutes of Health, Bethesda, MD 20894, USA

## Abstract

**Background:**

An evolutionary classification of genes from sequenced genomes that distinguishes between orthologs and paralogs is indispensable for genome annotation and evolutionary reconstruction. Shortly after multiple genome sequences of bacteria, archaea, and unicellular eukaryotes became available, an attempt on such a classification was implemented in Clusters of Orthologous Groups of proteins (COGs). Rapid accumulation of genome sequences creates opportunities for refining COGs but also represents a challenge because of error amplification. One of the practical strategies involves construction of refined COGs for phylogenetically compact subsets of genomes.

**Results:**

New Archaeal Clusters of Orthologous Genes (arCOGs) were constructed for 41 archaeal genomes (13 Crenarchaeota, 27 Euryarchaeota and one Nanoarchaeon) using an improved procedure that employs a similarity tree between smaller, group-specific clusters, semi-automatically partitions orthology domains in multidomain proteins, and uses profile searches for identification of remote orthologs. The annotation of arCOGs is a consensus between three assignments based on the COGs, the CDD database, and the annotations of homologs in the NR database. The 7538 arCOGs, on average, cover ~88% of the genes in a genome compared to a ~76% coverage in COGs. The finer granularity of ortholog identification in the arCOGs is apparent from the fact that 4538 arCOGs correspond to 2362 COGs; ~40% of the arCOGs are new. The archaeal gene core (protein-coding genes found in all 41 genome) consists of 166 arCOGs. The arCOGs were used to reconstruct gene loss and gene gain events during archaeal evolution and gene sets of ancestral forms. The Last Archaeal Common Ancestor (LACA) is conservatively estimated to possess 996 genes compared to 1245 and 1335 genes for the last common ancestors of Crenarchaeota and Euryarchaeota, respectively. It is inferred that LACA was a chemoautotrophic hyperthermophile that, in addition to the core archaeal functions, encoded more idiosyncratic systems, e.g., the CASS systems of antivirus defense and some toxin-antitoxin systems.

**Conclusion:**

The arCOGs provide a convenient, flexible framework for functional annotation of archaeal genomes, comparative genomics and evolutionary reconstructions. Genomic reconstructions suggest that the last common ancestor of archaea might have been (nearly) as advanced as the modern archaeal hyperthermophiles. ArCOGs and related information are available at: .

**Reviewers:**

This article was reviewed by Peer Bork, Patrick Forterre, and Purificacion Lopez-Garcia.

## Background

A robust classification of genes based on accurately deciphered evolutionary relationships is the cornerstone of comparative and evolutionary genomics. Such a classification is indispensable both for the functional annotation of sequenced genomes and for any genome-wide evolutionary reconstruction. The construction of an evolutionary classification of genes is a non-trivial task because of the complexity of homologous relationships between genes. The two principal classes of homologs are orthologs and paralogs. Orthologs are homologous genes that evolved via vertical descent from a single ancestral gene in the last common ancestor of the compared species. Paralogs are homologous genes, which, at some stage of evolution, have evolved by duplication of an ancestral gene [1,2]. Orthology and paralogy are intimately linked because, if a duplication (or a series of duplications) occurs after the speciation event that separated the compared species, orthology becomes a relationship between sets of paralogs, rather than individual genes (in which case, such genes are called co-orthologs).

Correct identification of orthologs and paralogs is of central importance for both the functional and the evolutionary aspects of comparative genomics. Orthologs typically occupy the same functional niche in different organisms; by contrast, paralogs evolve to functional diversification as they diverge after the duplication [3,4]. Therefore, the accuracy of genome annotation critically depends on the accurate identification of orthologs [5]. A clear demarcation of orthologs and paralogs is also required for constructing evolutionary scenarios which include, along with vertical inheritance, lineage-specific gene loss and horizontal gene transfer (HGT) [6-8].

In principle, orthologs, including co-orthologs, should be identified by means of phylogenetic analysis of entire families of homologous proteins in the compared genomes, which is expected to define orthologous protein sets as clades. However, for genome-wide protein sets, such analysis remains extremely labor-intensive, and error-prone as well [9]. Accordingly, procedures have been developed for identification of sets of likely orthologs without an explicit referral to phylogenetic analysis. These procedures are based on the notion of a genome-specific best hit (BeT), i.e., the protein from a target genome that is most similar (typically, in terms of similarity scores computed using BLAST or another sequence comparison method) to a given protein from the query genome [10,11]. The assumption central to this approach is that orthologs have a greater similarity to each other than to any other protein from the respective genomes. When multiple genomes are analyzed, pairs of probable orthologs detected on the basis of BeTs are combined into orthologous clusters represented in all or a subset of the analyzed genomes. This approach, amended with additional procedures for detecting co-orthologous protein sets and for treating multidomain proteins, was implemented in the database of Clusters of Orthologous Groups (COGs) of proteins [11,12]. The latest COG set released in 2003 includes ~70% of the proteins encoded in 69 genomes of prokaryotes and unicellular eukaryotes [13]. The COGs have been employed for functional annotation of newly sequenced genomes (e.g. [14,15], comparative analysis of gene neighborhoods [16-18] and other types of connections between genes, as implemented in the widely used STRING tool [19], target selection in structural genomics (e.g. [20], and various genome-wide evolutionary analyses [7,8]. Independently, other groups have developed similar methodologies for identification of orthologs and paralogs in pairwise or multiple genome comparisons [21,22]. Very recently, a major effort on automatic construction of sets of orthologous genes has culminated in the EggNOG database which employed the COGs as a prototype and a seed [23].

The methods for the construction of COGs were developed and originally applied to small sets of genomes; these and other related methods do not guarantee correct identification of the paralogous and orthologous relationships, due to the variability of domain architectures of proteins, differential loss of paralogs in different lineages, extreme divergence of some orthologous and paralogous genes, and other complications [2,12,13]. The computational cost of exhaustive genome comparisons also grows almost prohibitively with the steep increase in the number of sequenced genomes which approached 500 in the beginning of 2007 [24]. Thus, several smaller scale studies have been conducted in which COGs were constructed for compact groups of bacteria including the *Thermus-Deinococcus *group [25], *Cyanobacteria *[26], and *Lactobacillales *[15]. In each of these analyses, a considerably better resolution of the homologous relationship than in the overall COG set has been achieved.

In the previous comparative-genomic analyses of archaea, we delineated COGs for this domain of life and used them to partition archaeal genes into the evolutionarily stable, conserved core and the "shell" of genes that are often lost during evolution or are characteristic of a narrow group of species [27]; we further traced the dynamics of drop in the number of the core genes with sequencing of additional archaeal genomes [28,29].

Here we present the updated set of COGs that includes 41 sequenced archaeal genomes and delineate the core sets of genes that are represented in all archaea or in the major archaeal divisions, Euryarchaeota and Crenarchaeota. We further describe evolutionary reconstructions aimed at inferring the nature of the Last Archaeal Common Ancestor (LACA) and other ancestral forms, and uncovering the trends of gene loss and gain during archaeal evolution.

## Results and Discussion

### The archaeal genomic data set and construction of archaeal COGs

Table 1 lists the basic features of the analyzed archaeal genomes. The now available set of genomes represents reasonably well the genomic, taxonomic, and ecological diversity of archaea. The genome span the range from ~0.58 Mb (the parasite *Nanoarchaeum equitans*) to ~5.8 Mb (the mesophilic euryarchaeon *Methanosarcina acetivorum*); there are 20 hyperthermophiles and 21 mesophiles and moderate thermophiles; 27 genomes represent the Euryrchaeota, 13 belong to the Crenarchaeota, and the remaining one is *N. equitans *whose taxonomic position is considered uncertain [30,31].

**Table 1:** The 41 archaeal genomes included in the arCOGs

*Species*	Division	Lineage	Abbreviation	Genome size, Mb	Number of annotated protein-coding genes	OGT^a^	Life style and other features	Ref^b^	GenBank accession
*Aeropyrum pernix*	Crenarchaeota	Desulfurococcales	Aerpe	1.7	1700	90°C	Aerobic chemorganotroph, sulfur enhances growth	[60]	BA000002.3
*Caldivirga maquilingensis *IC-167	Crenarchaeota	Thermoproteales	Calma	2	1943	90°C	Moderate acidophile, heterotroph, anaerobe or microaerophyle		AAXQ00000000
*Cenarchaeum symbiosum*	Crenarchaeota	Cenarchaeales	Censy	2	2017	~10°C	Moderate psychrophile, uncultivated symbiont of sponges	[33]	DP000238
*Hyperthermus butylicus*	Crenarchaeota	Desulfurococcales	Hypbu	1.7	1602	>100°C	Hyperthermophilic neutrophile, anaerobe	[61]	CP000493.1
*Pyrobaculum aerophilum*	Crenarchaeota	Thermoproteales	Pyrae	2.2	2605	100°C	Facultative nitrate-reducing anaerobe	[62]	AE009441.1
*Pyrobaculum calidifontis *JCM 11548	Crenarchaeota	Thermoproteales	Pyrca	2	2149	100°C	Same as Pyrae	NA	CP000561.1
*Pyrobaculum islandicum *DSM 4184	Crenarchaeota	Thermoproteales	Pyris	1.8	1978	100°C	Same as Pyrae	NA	CP000504.1
*Staphylothermus marinus *F1	Crenarchaeota	Desulfurococcales	Stama	1.6	1570	80°C	Anaerobic submarine heterotroph	NA	CP000575.1
*Sulfolobus acidocaldarius *DSM 639	Crenarchaeota	Sulfolobales	Sulac	2.2	2223	80°C	Aerobic thermoacidophile	[63]	CP000077.1
*Sulfolobus solfataricus*	Crenarchaeota	Sulfolobales	Sulso	3	2977	80°C	Sulfur-metabolizing chemorganotroph, thermoacidophilic, motile aerobe	[64]	AE006641.1
*Sulfolobus tokodaii*	Crenarchaeota	Sulfolobales	Sulto	2.7	2825	80°C	Same as Sulso	[65]	BA000023.2
*Thermofilum pendens *Hrk 5	Crenarchaeota	Thermoproteales	Thepe	1.8	1876	92°C	Acidophilic anaerobe	NA	CP000505.1
*Thermoproteus tenax*	Crenarchaeota	Thermoproteales	Thete	1.8	2021	96°C	Facultative hydrogen-sulfur authotroph, anaerobe	NA	n/a
*Archaeoglobus fulgidus*	Euryarchaeota	Archaeoglobales	Arcfu	2.2	2420	83°C	Motile, anaerobic, sulfate-reducing chemolito- or chemorgano- autothroph	[66]	AE000782.1
*Haloarcula marismortui *ATCC 43049	Euryarchaeota	Halobacteriales	Halma	4.3	4240	37°C	Chemoorganotrophic obligate halophile	[67]	AY596297.1
*Halobacterium sp*	Euryarchaeota	Halobacteriales	Halsp	2.6	2622	37°C	Aerobic chemorganotroph, obligate halophile, proteolytic, motile, with cell envelope; 2 extrachromosomal elements	[68]	AE004437.1
*Haloquadratum walsbyi*	Euryarchaeota	Halobacteriales	Halwa	3.2	2646	37°C	Halophilic, aerobic heterotroph	[69]	AM180088.1
*Methano-thermobacter thermo-autotrophicus*	Euryarchaeota	Methanobacteriales	Metth	1.8	1873	65°C	Chemolitoautothroph, strict anaerobe, nitrogen-fixing methanogen	[70]	AE000666.1
*Methanococcoides burtonii *DSM 6242	Euryarchaeota	Methanosarcinales	Metbu	2.6	2273	23°C	Psychrotolerant, strictly anaerobic, slightly halophilic methylotroph	NA	CP000300.1
*Methanocaldo-coccus jannaschii*	Euryarchaeota	Methanococcales	Metja	1.7	1786	85°C	Chemolito-autothrophic, strictly anaerobic, motile methanogen, 2 extrachromosomal elements	[71]	L77117.1
*Methanococcus maripaludis *C5	Euryarchaeota	Methanococcales	MetmC	1.8	1822	37°C	Mesophilic hydrogenotrophic, nitrogen-fixing methanogen	[72]	CP000609.1
*Methanococcus maripaludis *S2	Euryarchaeota	Methanococcales	Metmp	1.7	1722	37°C	same as MetmC	NA	BX950229.1
*Methanocorpusculum labreanum *Z	Euryarchaeota	Methanomicrobiales	Metla	1.8	1739	37°C	Strictly anaerobic, CO_2 _fixing methanogen	NA	CP000559.1
*Methanoculleus marisnigri *JR1	Euryarchaeota	Methanomicrobiales	Metcu	2.5	2489	37°C	Strictly anaerobic methanogen	NA	CP000562.1
*Methanopyrus kandleri*	Euryarchaeota	Methanopyrales	Metka	1.7	1687	110°C	Chemolito-autothrophic, strictly anaerobic, methanogen, high intracellular salt concentration	[41]	AE009439.1
*Methanosaeta thermophila *PT	Euryarchaeota	Methanosarcinales	Metsa	1.9	1696	60°C	Strictly anaerobic methanogen	NA	CP000477.1
*Methanosarcina acetivorans*	Euryarchaeota	Methanosarcinales	Metac	5.8	4540	37°C	Chemolito-autothrophic, anaerobic, nitrogen-fixing, versatile methanogen, motile, forms multicellular structures	[73]	AE010299.1
*Methanosarcina barkeri fusaro*	Euryarchaeota	Methanosarcinales	Metba	4.8	3624	37°C	Same as Mac	[74]	CP000099.1
*Methanosarcina mazei*	Euryarchaeota	Methanosarcinales	Metma	4.1	3370	37°C	Same as Mac	[75]	AE008384.1
*Methanosphaera stadtmanae*	Euryarchaeota	Methanobacteriales	Metst	1.8	1534	37°C	Methanogen, human intestinal inhabitant	[76]	CP000102.1
*Methanospirillum hungatei *JF-1	Euryarchaeota	Methanomicrobiales	Methu	3.5	3139	37°C	Strictly anaerobic methanogen	NA	CP000254.1
*Natronomonas pharaonis*	Euryarchaeota	Halobacteriales	Natph	2.8	2822	37°C	Extreme haloalkaliphile	[77]	CR936257.1
*Picrophilus torridus *DSM 9790	Euryarchaeota	Thermoplasmales	Picto	1.6	1535	65°C	Extremely acidophilic moderate thermophile	[78]	AE017261.1
*Pyrococcus abyssi*	Euryarchaeota	Thermococcales	Pyrab	1.8	1898	96°C	Same as Pho	[79]	AL096836.1
*Pyrococcus furiosus*	Euryarchaeota	Thermococcales	Pyrfu	1.9	2125	96°C	Same as Pho	[80]	AE009950.1
*Pyrococcus horikoshii*	Euryarchaeota	Thermococcales	Pyrho	1.7	1955	96°C	Anaerobic, motile heterotroph	[81]	BA000001.2
*Thermococcus kodakaraensis *KOD1	Euryarchaeota	Thermococcales	Theko	2.1	2306	85°C	Anaerobic heterotroph	[82]	AP006878.1
*Thermoplasma acidophilum*	Euryarchaeota	Thermoplasmales	Theac	1.6	1482	59°C	Chemorganotrophic, thermoacidophilic, motile facultative anaerobe	[83]	AL139299.1
*Thermoplasma volcanium*	Euryarchaeota	Thermoplasmales	Thevo	1.6	1499	60°C	Same as Tac	[84]	BA000011.4
Uncultured methanogenic archaeon	Euryarchaeota	?	Uncme	3.2	3085	37°C	Methanogen isolated from rice rhizosphere	NA	AM114193.2
*Nanoarchaeum equitans*	Nanoarchaeota	?	Naneq	0.5	536	80°C	Obligate symbiont of the crenarchaeon *Ignicoccus*	[30]	AE017199.1

^a^OGT, optimal growth temperature

^b^Only the references that report the complete genome of the respective species and its initial analysis are cited

**Figure 1:**
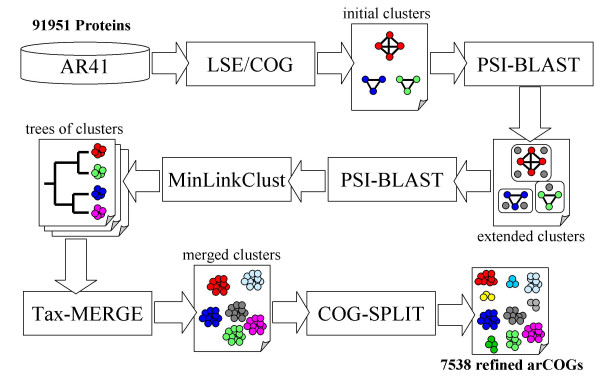
A flow chart of the procedure employed for the construction of the arCOGs. See Materials and Methods for the description of each step.

The archaeal COGs (arCOGs) were constructed using a new computational pipeline (Fig. 1) which is a substantial modification of the previously published procedures [11,13]. Briefly, the pipeline consists of the initial step of the all-against-all comparison of the protein sequences encoded in the analyzed genomes; preliminary clustering to identify lineage-specific expansions (LSEs) of paralogs, genes that are inferred to have evolved by duplication after the divergence of the compared species; delineation of clusters of bidirectional best hits to form initial, crude COGs; iterative search of the rest of the archaeal protein databases to accrue potential diverged members of the COGs; minimum linkage clustering of the COGs; merge of related COGs with supplementary phyletic patterns to avoid oversplitting of fast evolving COGs; splitting of potentially overclumped COGs; the details of each of these procedures are given under Materials and Methods.

### Coverage of archaeal genomes with arCOGs

**Figure 2:**
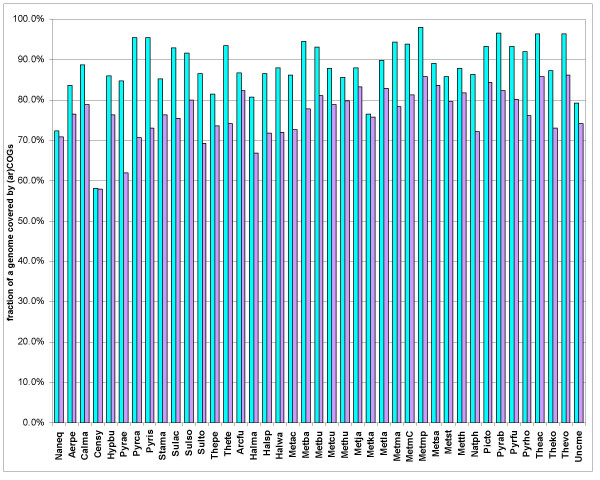
Coverage of archaeal genomes with arCOGs and COGs. Cyan, ArCOGs, purple, COGs. Abbreviations are as in Table 1.

Altogether, the process of arCOGs construction started with 91,951 proteins encoded in 41 archaeal genomes and ended with 80,963 of these proteins being included in 7,538 arCOGs (the arCOGs and accompanying materials are available online [32]). The fraction of the proteins encoded in a genome that belong to the COGs is a crucial number in the comparative-genomic analysis as it characterizes both the level of conservation and coherence between the analyzed genomes, and the potential for genome annotation by inference from homology. Already in the early COG analyses, with a small number of genomes included, it has been noticed that a substantial majority of the genes had orthologs in other genomes [12]. With the growth of the genome collection and the new, refined procedure for COG construction, the coverage of archaeal genomes further increased. Figure 2 shows that, on average, the arCOGs described here cover 88% of the genes in an archaeal genome as compared to 76% with the previous release of COGs which included 69 genomes, among these, 13 archaea (for this comparison, the proteins from the 41 analyzed archaeal genomes were fit into the old COGs using the COGNITOR program). Predictably, the extra coverage was most pronounced for genomes that had close relatives within the analyzed set such as *Halobacteria*, *Pyrobaculi*, and *Methanosarcina*, but a substantial increase was seen across the entire set of genomes, with two notable exceptions, *Nanoarchaeum equitans *and, particularly, *Cenarchaeum symbiosum *(Fig. 2). In the case of *C. symbiosum*, somewhat paradoxically, the coverage with the old COGs was even somewhat greater than with the new arCOGs (Fig. 2). The reasons behind the poor coverage of these two genomes are clear. Both *N. equitans *and *C. symbiosum *have no close relatives in the current collection of archaeal genomes; in addition, *N. equitans *appears to be a very fast-evolving lineage [31] whereas *C. symbiosum *is a symbiotic crenarchaeon that seems to have acquired lots of bacterial genes and possesses a number of unique gene families [33], which leads to a poor representation in ar COGs (see also below). In addition to the increased coverage, the new arCOGs appear to provide a better resolution of orthologous relationships than the existing COG set: 719 of the old COGs [13] were split into two or more arCOGs, and the mean number of genes from an archaeal genome per COG (species-specific paralogs) dropped from 1.65 in the COGs (36% clusters with no paralogs) to 1.34 in arCOGs (58% clusters with no paralogs).

### Phyletic patterns, conserved cores and variable shells of archaeal genomes

**Figure 3:**
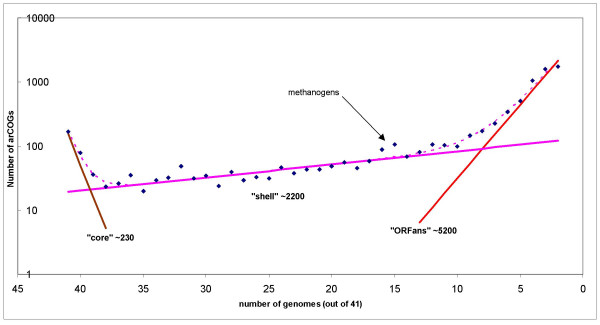
Distribution of the number of species in arCOGs: three classes of archaeal genes. A semi-logarithmic plot fitted with a sum of 3 exponents

In an early comparative-genomic study of the archaea, we developed the notion of a conserved core of genes that are shared by all or the substantial majority of the genomes and, by inference, are likely to be essential for the cell function, as opposed to the variable "shell" of genes that show diverse distributions among species and, accordingly, appear to be subject to lineage-specific gene loss and horizontal gene transfer (HGT) [27]. The current analysis of a much larger collection of archaeal genomes provides for a refinement of these concepts. Figure 3 shows the distribution of the number of archaeal species in the arCOGs. Obviously, in quantitative terms, arCOGs with a small number of species (<6) dominate the collection; the distribution is, essentially, an exponential decay curve, with a rise at the left end (40 or 41 species), which corresponds to the archaeal genomic core (see below), and a bump at 15 species which correspond to the 15 available genomes of methanogens. More formally, assuming that the distribution is described by an exponent(s), the best approximation is achieved with a sum of three exponential functions (Fig. 3). The first exponent can be construed to represent the conserved gene core (~230 arCOGs), the second one describes the "shell" of moderately common genes (~2200 arCOGs), and the third one corresponds to the "ORFans" (~5200 arCOGs), which include a small number of (typically, but not necessarily, closely related) species.

**Figure 4:**
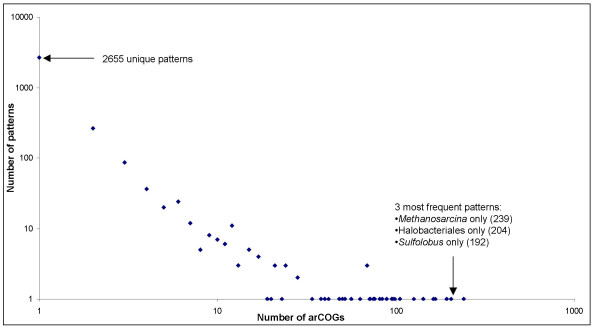
Distribution of phyletic patterns by the number of arCOGs. A log-log plot.

The notion of a phyletic pattern which is, simply, the pattern of presence-absence of a COG in the analyzed set of a species, has been developed in the original COG study [11]and, independently, by others [34]. Subsequently, phyletic patterns have been extensively employed both for functional prediction and as starting material for evolutionary reconstruction (e.g. [7,8,35-38]). Figure 4 shows the distribution of the phyletic patterns in the new set of arCOGs. The decay of the curve is remarkably steep, i.e., a substantial majority of the patterns (2654 of 3192) are unique, that is, represented by one arCOG only. Examination of the list of the top 10 widespread arCOGs is particularly instructive (Table 2). In this list, 9 patterns are "trivial", i.e., represented in multiple species of a compact monophyletic group, such as Methanosarcinales or Halobacteriales. The single exception is the "all" pattern which describes the strictly defined core of 165 archaeal genes represented in all currently sequenced genomes. The most common relatively "non-trivial" pattern is the one that includes arCOGs represented in all species except for *N. equitans *(50 arCOGs); again, this is hardly unexpected given the small number of genes in *N. equitans*, suggesting massive gene loss. Although phyletic patterns provide only a crude assessment of the relationship between the compared genomes and caution is due, such that too sweeping conclusions on evolution are not drawn solely from the inspection of these patterns, some conjectures from the trend seen in Figure 4 and in Table 2 appear straightforward. The uniqueness of most of the phyletic patterns suggests that emergence of new families in individual lineages, lineage-specific gene loss, and HGT are all major forces of archaeal evolution. However, the absence of common non-trivial patterns suggests that distinct "highways" of HGT [39]do not shape archaeal evolution.

**Table 2:** The 10 most common phyletic patterns in the arCOGs

Lineage	Species^a^	Number of arCOGs
Mathanosarcinales	Metac, Metba, Metma	239
Halobacteriales	Halma, Halsp, Halwa, Netph	204
Sulfolobales	Sulac, Sulso, Sulto	192
**All**	**All 41**	**166**
Thermoproteales	Pyrae, Pyrca, Pyris, Thete	162
Thermococcales	Pyrab, Pyrfu, Pyrho, Theko	142
Methanosarcinales	Metac, Metba	126
Methanococcales	MetmC, Metmp	105
Halobacteriales	Halma, Halwa	99
Thermoplasmales	Picto, Theac, Thevo	96

^a^Abbreviations are as in Table 1.

**Figure 5:**
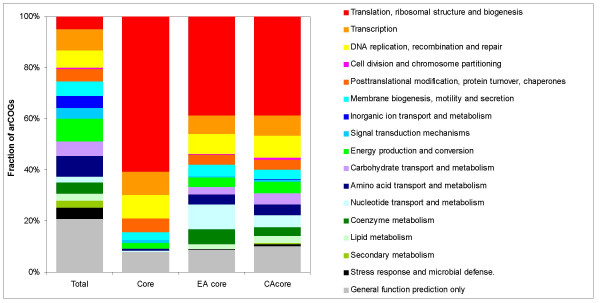
Functional breakdown of the entire set of arCOGs and the three core sets. EA, Euryarchaea, CA, Crenarchaea.

The 166 arCOGs that comprise the strictly defined core of the archaeal genomes, as well as the core gene sets of Euryarchaeota and Crenarchaeota, are, for obvious reasons, of special interest. First, it has to be noticed that the euryarchaeal core (282 arCOGs) and the crenarchaeal core (336 arCOGs) are not dramatically larger than the pan-archaeal core, emphasizing the high prevalence of gene loss and gain (on many occasions, via HGT) during the evolution of archaeal genomes. Along the same lines, but more unexpectedly, the euryarchaeal and crenarchaeal genomic signatures, i.e., the sets of arCOGs that are represented in all species in one group but not found in any species of the other group, consist of only one and three arCOGs, respectively. In agreement with previous observations, a breakdown of the functional assignments (according to the broad functional categories associated with the COGs [13]) reveals dramatic differences between the overall set of arCOGs and the core sets (Fig. 5). Indeed, each of the core sets is dominated by proteins functioning in the translation system, ribosome biogenesis, and tRNA modification, with additional significant contributions from other information-processing systems (basal transcription and replication). Moreover, even for the few core archaeal genes that remain experimentally uncharacterized, roles in translation and RNA modification could be predicted on the basis of the analysis of domain organization and operonic context (see [32]; KSM and EVK, unpublished). In a stark contrast, in the overall arCOG set, the informational functions are quantitatively minor whereas metabolic functions are dominant (Fig. 5).

### Applications of arCOGs for evolutionary genomics of archaea: gene-content tree, evolutionary reconstructions, and putative phylogenetic of core and shell genes

From the inception of the COG methodology, it had been realized that COGs have potential for straightforward evolutionary-genomic applications. One of these is the construction of gene-content trees whereby the phyletic patterns of COGs are converted into a distance matrix between the analyzed genomes, with an appropriate normalization for genome size [37,38,40](see Materials and Methods).

**Figure 6:**
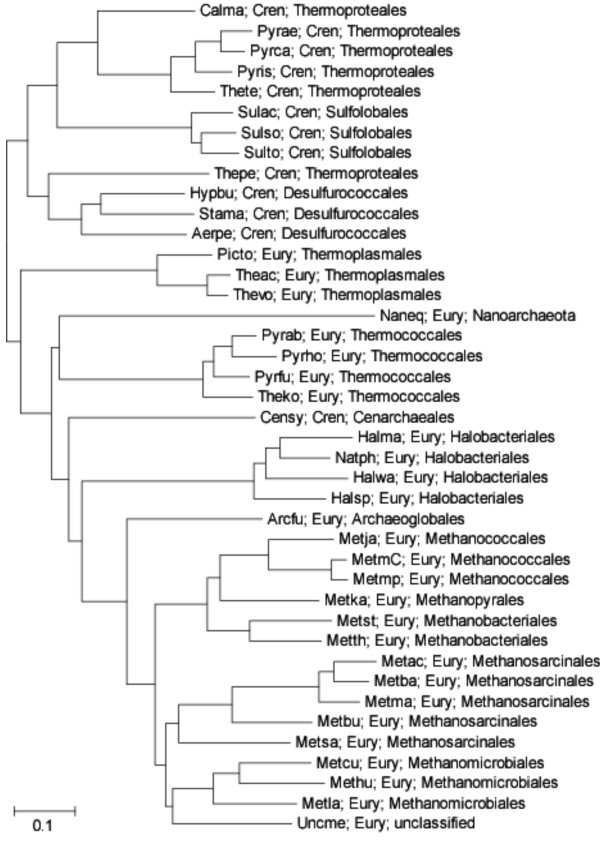
The gene-content tree of archaea constructed on the basis of the phyletic patterns of arCOGs. The species abbreviations are as in Table 1. Cren, Crenarchaeota; Eury, Euryarchaeota.

We used the phyletic patterns of the arCOGs as the input to produce a gene-content tree for the 41 analyzed archaeal species. Gene-content trees are known to reflect combination of bona fide phylogenetic relationships, horizontal gene flows, and life style differences between organisms leading to parallel gene loss. It appears that the archaeal gene-content tree carries a substantial phylogenetic signal (Fig. 6). The tree supports the major phylogenetic divisions within the archaea, i.e., the monophyly of Euryarchaeota and Crenarchaeota, and most of the branches within each of these divisions. However, at least three aspects of this tree deserve special attention. Firstly, the tree has methanogens as a clade within the Euryarchaeota. Regular, sequence-based phylogenetic analyses tend to break the methanogens into two or three clades, namely, methanococcales-methanothermobacteriales, methanosarcinales (typically, joined with halobacteriales), and *Methanopyrus kandleri*. The phylogenetic position of *M. kandleri *remains uncertain although monophyly with *Methanococcales *and *Methanobacteriales *is likely [41,42], the placement of *Methanosarcinales *apart from the rest of the methanogens appears to be solidly supported [42]. Most likely, the aggregation of the methanogens in the gene-content tree in Fig. 6 is caused by the shared genes encoding proteins involved in methanogenesis which might have spread both vertically and horizontally. Secondly, *N. equitans *is placed deeply within the *Thermococcales *branch. The initial phylogenetic analysis has been interpreted to indicate that this unusual organism was a basal, ancestral archaeal branch [30]. However, a subsequent reappraisal suggested that the basal position of *N. equitans *was a long branch attraction artifact, and the correct placement of *N. equitans *should be with the *Thermococcales *[31]; remarkably, the tree content analysis is best compatible with this hypothesis. Thirdly, the single available genome of a mesophilic crenarchaeon, *C. symbiosum*, falls within the euryarchaeal part of the tree; the definitive resolution of the phylogenetics affinities of mesophilic Crenarchaeota requires a representative collection of genomes but the present observations already indicate that *C. symbiosum *is not a typical crenarchaeon. At least, in part, this position of *C. symbiosum *in the gene-content tree could be explained by acquisition of euryarchaeal genes via HGT [43].

**Figure 7:**
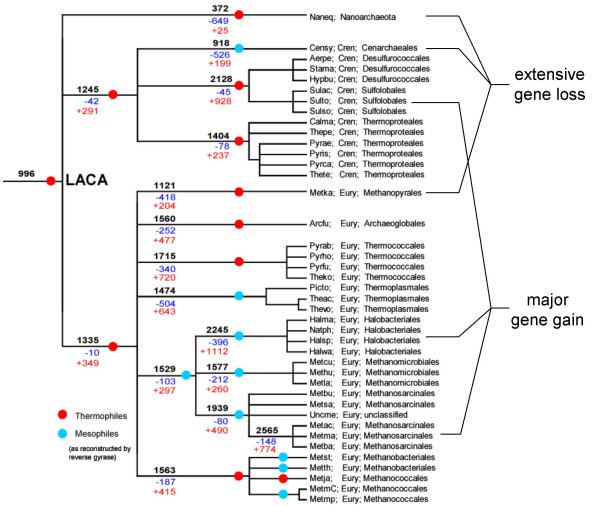
A reconstruction of gene gain and loss in archaea. Each branch is labeled by 3 numbers: black, the (inferred) number of arCOGs in the node to which the given branch leads; blue, number of arCOGs lost along the branch; red, number of arCOGs gained along the branch. The red circles on branches denote hyperthermophiles, and blue circles denote mesophiles and moderate thermophiles.

We then addressed the reverse problem, namely, reconstruction of the history of gene gain and loss in archaea given a particular phylogenetic tree topology. On the account of the uncertainty of the deep branches in archaeal phylogeny, we chose to use a partially unresolved tree in which the relationship between several clades is presented as a multifurcation (Fig. 7). The reconstruction was, then, performed using a modification of the weighted parsimony method [7] that has been previously applied to the analysis of the evolution of several major groups of bacteria [15,25,26]. With all the caveats in mind, the results of this reconstruction reveal notable trends of gene gain and loss in different lineages of archaea as well as features of the inferred ancestral forms. Predictably, the most massive gene loss is seen in *N. equitans *(a parasite with the smallest known genome among archaea), closely followed by *Thermoplasmales *(another group of heterotrophic archaea with small genomes) and *C. symbiosum *(a symbiotic archaeon that might have undergone a major life style shift). The lineages with the most gene gain include those with the largest genomes, namely, *Halobacteriales *and *Methanosarcinales*, and *Sulfolobales-Desulfurococcales*. More unexpectedly, substantial gene gain, along with major gene loss, was inferred also for *Thermococcales *and *Thermoplasmales*; apparently, these are groups with highly dynamic genomes.

**Figure 8:**
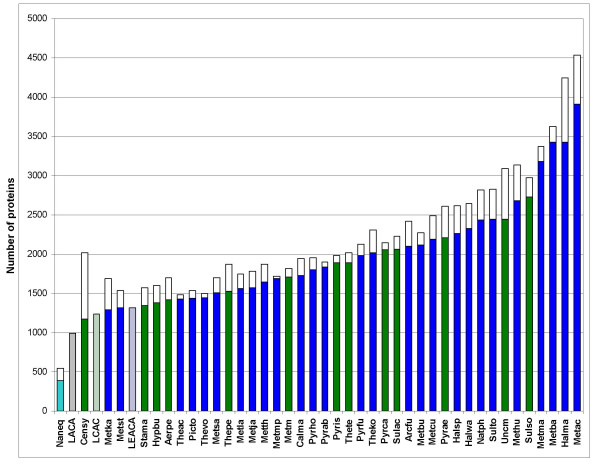
Low-bound reconstructions for ancestral archaeal forms: genomes close in size to modern hyperthermophiles. Each column shows the total number of annotated protein-coding genes in the respective archaeal species; the colored portions (green for Crenarchaeota, blue for Euryarchaeota, and cyan for Nanoarchaeota) show genes included in arCOGs. The hatched columns show the number of arCOGs assigned to LACA, the Last CrenArchaeal Common Ancestor (LCACA) and the Last EuryArchaeal Common Ancestor (LEACA).

The present reconstruction maps almost 1000 archaeal genes to LACA and ~1300 and ~1400 genes to the ancestors of Crenarchaeota and Euryarchaeota, respectively (Fig. 7). These numbers are notable in that the ancestral gene sets appear to be not much smaller than the smallest genomes of the extant free-living archaea, such as *Thermoplasma *(Figs. 7 and 8). It must be kept in mind that these numbers are low bounds for the gene content of the ancestral forms inasmuch as parsimony has a fundamental bias toward underestimating the amount of gene loss [44]. Thus, perhaps, unexpectedly, it appears that ancestral forms, including LACA, were not much simpler, at least in terms of genomic complexity but, most likely, also in their cellular organization than some modern forms. This conjecture is further supported by a more detailed examination of the genes (arCOGs) assigned to LACA (Table 3). In particular, the results of the reconstruction suggest that LACA was a hyperthermophile that possessed reverse gyrase, the principal hallmark of the hyperthermophilic lifestyle [45] and most of the other genes characteristic of hyperthermophiles ([36] and unpublished observations), and a chemoautotroph that had the genes to support membrane-based redox bioenergetics and all central biosyntheses (Table 3). Notably, the reconstruction also indicates that LACA already possessed some widespread functional systems of archaea that are not normally thought of as being ancestral including the CASS system of antiviral defense [46,47] and the predicted toxin-antitoxin system centered around the "minimal" nucleotidyltransferases ([48] and KSM, YIW, and EVK, unpublished).

**Table 3:** Major features of the reconstructed gene set of LACA

**COG class**	**No. of arCOGs**	**Function**	**Implication for LACA**
**J**	**152**	**Translation**	Complete translation system and essentially complete set of enzymes for tRNA and rRNA modification
including:	61	Ribosomal proteins	
	21	aaRS and related enzymes	
**K**	**55**	**Transcription**	Moderately sophisticated transcription control
including:	22	Transcription regulators	
	13	RNA polymerase subunits	
**L**	**61**	**Replication, recombination and repair**	Advanced DNA replication and repair system
including:	6	Topoisomerases	
	4	DNA polymerase subunits	
**C**	**84**	**Energy production and conversion**	Membrane-based redox bioenergetics; partial TCA cycle
including:	13	Pyruvate oxidation	
	9	TCA cycle	
	9	NADH dehydrogenase or Na+/H+ antiporter	
	8	V-type ATPase-ATP synthase	
**G**	**33**	**Carbohydrate transport and metabolism**	Moderately sophisticated sugar metabolism
including:	8	Glycolysis/Gluconeogenesis	
**E**	**108**	**Amino acid transport and metabolism**	Enzymes for the biosynthesis of all amino acids
including:	72	Amino acid biosynthesis	
**F**	**49**	**Nucleotide transport and metabolism**	Enzymes for the biosynthesis of all nucleotides
including:	29	Nucleotide biosynthesis	
	6	Nucleotide salvage	
**H**	**67**	**Coenzyme transport and metabolism**	Enzymes for the biosynthesis of all essential cofactors
including:	60	Cofactor biosynthesis	
**I**	**25**	**Lipid transport and metabolism**	Fully developed membrane
including:	19	Lipid biosynthesis	
**M**	**26**	**Cell wall, membrane and envelope biogenesis**	Fully developed cell wall
**P**	**48**	**Inorganic ion transport and metabolism**	Sophisticated ion uptake system
**Q**	**8**	**Secondary metabolites biosynthesis, transport and catabolism**	Limited or unknown
**N**	**5**	**Cell motility**	Limited motility and/or conjugation
**O**	**47**	**Posttranslational modification, protein turnover, chaperones**	Sophisticated system of protein fate control
including:	2	Proteasome	
**D**	**5**	**Cell cycle control**	Limited or unknown
**T**	**10**	**Signal transduction mechanisms**	Limited use of bacterial type signal transduction system; original signal transduction
including:	3	Serine/threonine kinase	
**U**	**10**	**Intracellular trafficking and secretion**	Fully developed secretion system
including:	3	Preprotein translocase	
**V**	**20**	**Defense mechanisms**	Viruses abundant at LACA times
including:	6	CASS proteins	
**R, S**	**183**	**Poorly characterized or unknown**	

**Figure 9:**
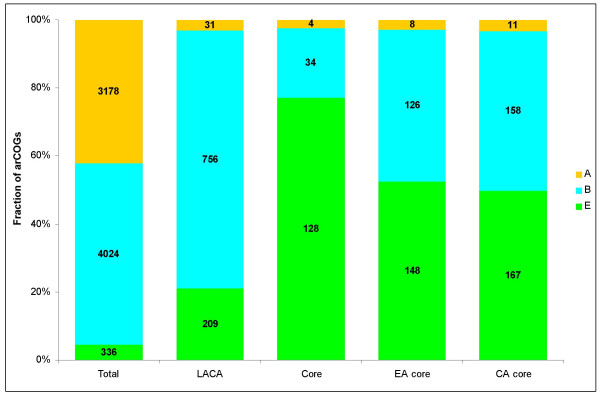
Taxonomic affinities of ArCOGs with bacteria and eukaryotes. For the criteria of taxonomic assignments, see Materials and Methods.A, archaea, B, bacteria, E, eukaryotes.

Finally, we attempted to obtain a crude breakdown of the phylogenetic affinities of the arCOGs, in particular, those that form the archaeal gene core (see above) and those that were assigned to LACA. Because a comprehensive phylogenomic analysis is beyond the scope of this paper (and has major potential for its own share of artifacts), this was done by analyzing the taxonomic breakdown of the proteins that are most similar to the representatives of a given arCOG as detected in BLAST searches. In order to eliminate potential effects of HGT, a special protocol was developed to identify coherent affinities, e.g., a bacterial affinity was assigned only when the proteins from a given arCOG had best hits in multiple bacterial species (see Materials and Methods). We are fully aware of the limitations of such methodology that, at best, gives a crude approximation of the true phylogeny [49] but we also note that this type of analysis can reveal highly meaningful patterns in comparative-genomic data (e.g. [50,51]). This analysis revealed striking differences between the overall set of arCOGs, LACA, and the core sets (Fig. 9). Clearly, the overall set is dominated by "bacterial" and archaea-specific genes, with a small fraction of "eukaryotic" genes; this fraction is somewhat greater in the inferred gene set of LACA but the dominance of "bacterial" genes remains obvious. The core sets, especially, the 166 genes shared by all sequenced archaeal genomes, present a stark contrast in that they are dominated by "eukaryotic" genes (Fig. 9).

Comparing these observations with those presented in Figs. 3 and 5, one comes to the conclusion that, quantitatively, archaeal genomes are dominated by the relatively mobile "shell" genes that belong to the common prokaryotic gene pool and encode the overwhelming majority of metabolic, structural, and signal transduction functions; a sharp contrast is presented by the stable, archaeo-eukaryotic core of information-processing genes. These quantitative conclusions, even if based on a crude analysis, are in a good agreement with the early observations on the bimodal distribution of the taxonomic affinities of archaeal genes [52], the subsequent observations on the affinities of eukaryotic genes [51,53], and the complexity hypothesis which posited distinct evolutionary fates of information and operational genes [54].

## Conclusion

The arCOGs, which are expected to be updated as genome sequencing progresses, are a resource for genome annotation of the newly sequenced archaeal genomes and the refinement of the existing annotations, as well as evolutionary reconstructions. Crude reconstructions presented here indicate that the ancestral archaeal forms, including LACA, probably, were full-fledged prokaryotes, of approximately the same level of complexity as the simplest of the modern free-living archaea.

## Methods

### Construction of archaeal COGs

Protein sets for 40 completely sequenced genomes of Archaea were downloaded from the NCBI FTP site [55] or from the RefSeq section of GenBank (*Caldivirga maquilingensis *IC-167, *Cenarchaeum symbiosum *and Uncultured methanogenic archaeon). Protein sequences of *Thermoproteus tenax *were kindly provided by Bettina Siebers with permission from the sequencing consortium. The procedure of COG construction involved the following steps.

1. All-against-all BLAST [56] search was used to establish the similarity relationships between the archaeal proteins. Lineage-specific expansions of paralogs were identified essentially as described previously [57,58]. Initial clusters based on triangles of symmetrical best hits were constructed using a modified COG algorithm [11,13]; the major difference in the current implementation was the strict symmetry requirement for the "best hit" relationship between proteins. This constraint lowers the number of false-positives but, in the presence of paralogs, leads to substantial underclustering [11]; this was rectified on the subsequent steps.

2. Multiple alignments of the initial cluster members were constructed using the MUSCLE program [59]; alignments were used to construct PSSMs for a PSI-BLAST search [56] against the database of Archaea proteins with the e-value threshold of 0.01; proteins (domains) were added to the corresponding best-scoring original clusters resulting in a set of expanded clusters.

3. Sequences of expanded cluster members were aligned using MUSCLE, and the PSSMs constructed from these alignment were used for a second round of PSI-BLAST search against the database of archaeal proteins. The search results were used to construct a similarity graph for the relationships between the expanded clusters. Formally, all statistically significant (e<0.01) hits in a search with the PSSM for a particular cluster were classified according to the cluster they belong to; clusters in the hit list were ranked according to the mean score across their members (members missing from the hit list were assigned an arbitrary score 2 bits below the significance threshold). An edge between the *i*-th and the *j*-th clusters was given weight equal to the lowest rank among the *i*→*j *and *j*→*i *relationships (i.e., if cluster *j *is the top-ranking hit when cluster *i *is the query but cluster *i *is the third-ranking hit for cluster *j*, then the edge connecting *i *and *j *is given the rank of 3). Connected components were extracted from the graph; pairs of nodes within a connected component were assigned an edge with a rank of infinity if they were not connected directly. A minimum-linkage clustering procedure was applied to the connected sets of clusters (if cluster *i *and *j *are merged, the edge between cluster *k *and the node, representing the merged clusters, is given the rank equal to the lowest rank of *k*-*i *and *k*-*j *edges), resulting in a rooted dendrogram of relationships between the clusters. Then each node on on the tree was labeled with the number of species that were present in all descendant clusters. Two rules were used to determine if the descendant clusters should be merged: i) if species-coverage of the node is at least 50% greater than that of any of the descendant nodes and ii) if, among the descendants of a node, one is species-rich and the other one is species-poor (formally, if *s*_*i*_>20*s*_*j*_/(10-*s*_*j*_) where *s*_*i *_and *s*_*j *_stand for the species-coverage of the species-rich and species-poor descendant nodes, respectively).

4. In parallel to the above procedures, a BLAST search against the COG 2003 database was performed, followed by using a modified COGNITOR program [11,13] to assign archaeal proteins to prokaryotic COGs. Merged clusters with proteins assigned to different COGs were split into COG-specific clusters to avoid clustering of paralogous proteins that previously have been assigned to different curated COGs.

### Reconstruction of gene gain and loss events during the evolution of Archaea

Reconstruction of gene gain and loss during the evolution of Archaea was performed using a modified weighted parsimony approach [7] implemented in a two-pass algorithm. First, a coarse-resolution multifurcating species tree was compiled from several single-gene phylogenetic reconstructions and taxonomic data. For each arCOG, the phyletic pattern indicating the presense/absence of the respective gene in each analyzed species was mapped onto the leaves of the tree. The first pass is performed in the leaves-to-root direction, and the number of descendant nodes containing the given gene is counted for each internal tree node. If this number is greater than or equal to the first (generally, more stringent) threshold, which is set for each node individually, the node is assigned state "1" (presence of the gene), otherwise it is assigned state "0" (absence of the gene). In the second pass, which is performed in the opposite, root-to-leaves direction, if the gene is absent in the given node (state "0") but present in its ancestor and the number of descendant nodes carrying this gene is greater than or equal to the second (generally more relaxed) threshold, the node is assigned state "1". For the guide tree and the thresholds, see [32].

## Reviewers' reports

### Reviewer 1: Peer Bork, European Molecular Biology Laboratory

The paper describes the construction of orthologous group for archea.

Given the success of the COGs and KOGs (a subset for eukaryotes with higher resolution) and the inability of current purely automatic procedures to produce reliable orthologus groups and, very importantly, their reliable functional annotation, I see this as an important resource for various studies. Furthermore, it uses a semi-automatic procedure that includes some clever guiding principles e.g. it takes into account phylogenetic gene presence patterns. The average coverage of 88% at a higher resolution than the current 76% COG coverage of genes in archeal genomes is another noteworthy and useful feature. As far as I can see, the arCOGs are of high quality and I look forward to use them.

There is no comparison to more recent orthology-built procedures, but I assume that this semi-automatic procedure presented here provides a more accurate picture than purely automatic methods.

The only concerns I have are availability/formate issues and some minimalistic Figure captions. Both should be easy to solve.

Taken together, I congratulate the authors for this nice, important and very useful piece of work.

**Authors' response:***The formats of the files on the ftp site were modified to increase transparency, and an extended README file was added. We hope this imporves accessibility which is, indeed, crucial. The figure captions were amended.*

### Reviewer 2: Patrick Forterre, Institut Pasteur and Université Paris-Sud

The «easy to use» COG database has been especially useful for the biological community. It has helped to improve the quality of genome annotation and has been widely adopted by non bioinformatic experts to perform preliminary rounds of comparative genomic analysis. The main problem with such popular database is the delay in their updating, a daunting task considering the current avalanche of completely sequenced genomes. The present paper by Kira Makarova and colleagues reports a much welcome update of the COG database that focus on archaea (arCOGs). The number of completely sequenced archaeal genomes remains quite low (compared to the situation with bacteria) allowing an exhaustive analysis that remains to be done for bacteria and eukarya. The arCOGs database will be for sure an extremely important source of information for the community working on archaea and for all scientists interested in comparative genomics and microbial evolution. The new analysis corresponds to a substantial increase in information compared to previous one, since around 40% of arCOGs are new.

In addition to the description of the arCOGs database, the paper by Kira Makarova and co-workers present several analyses that bring new (or update) data and raise several interesting evolutionary questions. In particular, they have built a gene-content tree based on the presence-absence of arCOGs in archaeal genome and estimated the evolution of the archaeal genome content along the evolutionary tree based on a gene loss and gain analysis. They reported several intriguing observations that are worth to be discussed in the framework of current debates on archaeal phylogeny and on the nature of the last universal archaeal ancestor.

Makarova and co-workers noticed that the number of strictly specific euryarchaeal and crenarchaeal proteins is very low (one and three, respectively). This seems to strongly argue in favour of the monophyly of Archaea (against the «eocyte» hypothesis). However, it should be interesting to present a slightly «relaxed» version of these cores, by allowing for the possibility for a protein to be missing in a group of related archaea (something quite frequently observed, for instance the lack of the euryarchaeal histone in Thermoplasmatales). More generally, it could be interesting in the future to define a category of conserved arCOGs (carCOGs?) present in all members of at least two archaeal orders in order to discriminate between ORFans arCOGs that are only present in one order (probably «recently» introduced by lateral gene transfer) and arCOGs of probable ancient origin that can tell us something about the evolutionary relationships between the diverse archaeal orders. It should be then interesting to determine if the distribution of such carCOGs correlate with the archaeal phylogeny based on various evolutionary markers.

The parasitic archaeon *Nanoarchaeum equitans *lacks the larger number (50) of universal arCOG, confirming that this archaeon probably evolved by «genome reduction». Some authors have suggested that *N. equitans *is a primitive organism. I suspect that there is a relatively high percentage of these 50 proteins that have homologues in Bacteria or Eukarya. This could be indicated as an argument in favour of the reduction scenario *versus *the "old nano" hypothesis! Interestingly, the gene content tree based on arCOGs groups *N. equitans *with Thermococcales among Euryarchaeota. Although gene-content trees can be sometimes highly biased by lateral gene transfer, this observation is in good agreement with a preliminary global analysis based on best BLAST-hits and refined phylogenies based on proteins of the small ribosomal subunits, reverse gyrase, Topo VI and elongation factors (Brochier et al.2005). This confirms that *N. equitans *should not be considered as a member of a new archaeal phylum (as already widely found in text-books!!) but as an odd member of the Euryarchaeota, probably, distantly related to Thermococcales.

Another puzzling observation is the grouping of *Cenarchaeum symbiosum *with euryarchaea in the gene-content tree. Interestingly, the COG coverage is quite similar for all archaeal genomes (around 88%) except for *C. symbiosum *and *N. equitans*. This can be explained by genome reduction in the case of *N. equitans*, but not in the case of *C. symbiosum *whose genome has a «normal» size. Significantly, the authors reported that the coverage of *C. symbiosum *genome with the old COGs was greater than with the new arCOGs! This indicates that this genome contains COGs present in Bacteria or *Saccharomyces cerevisiae *but not in any other archaeon. The proposed explanation is that *C. symbiosum *is a symbiotic crenarchaeon that has acquired lots of bacterial genes. An alternative hypothesis is that *C. symbiosum *is not a crenarchaeon after all, but represents an early branching archaeal phylum that contains bacterial and archaeal homologues that have been lost in other archaea.

From their reconstruction of gene loss and gain events, Makarova and co-workers suggest that the last Universal archaeal ancestor (LACA) was a hyperthermophile and a chemo-litoautotrophe with a minimal number of genes around 1000. They conclude that LACA might have been (nearly) as advanced as modern archaeal hyperthermophiles and found this conclusion quite «unexpected». I am not so surprised. It's a prejudice to think that ancestors are always simpler than present-day organisms and that ancient evolution always occurred toward more "complexity". There is no reason why reductive evolution, which has occurred so often in the evolution of modern cells, was not as pervasive in ancient time (Forterre and Philippe, 1999). In fact, an in-depth analysis of ribosomal protein distribution by Poch and co-workers already suggested a few years ago that the ribosome of LACA was probably more complex that the ribosome of any modern archaea (Lecompte et al., 2002).

**Authors' response: ***We do not, exactly, disagree and certainly realize the importance of reductive evolution. Still, whether or not we should consider the reconstruction of a complex LACA surprising or not, depends on the perspective. Considering that LACA is supposed to be the common ancestor of one of the 3 domains of life, there might be some element of surprise in this observation. After all, at the earliest stages of the evolution of life, there must have been a dramatic increase in complexity. That this complexification stage, apparently, was over by the time the domain of life became distinct (very likely, the same will hold for bacteria) is, certainly, of note. Alternatively, it is conceivable that LACA is actually not as ancient as one might think but represents a more recent bottleneck in archaeal evolution such that there was a complexification stage after the onset of the archaeal domain but it is inaccessible by comparative genomics*.

My only criticism of this paper is that the authors have taken a quite conservative view of archaeal phylogeny (only based on 16S rRNA) to analyse gene loss and gain along the archaeal history and to estimate the genome content of LUCA. Indeed, several features of their unresolved multifurcation tree are dubious.

*N. equitans *appears as an isolated lineages (a third phylum)

*C. symbiosum *is grouped with hyperthermophilic Crenarchaeota.

*Methanopyrus kandleri *is shown as an isolated branch

In all these cases, the authors have chosen to follow the 16S rRNA tree, whereas careful analyses based on ribosomal proteins have shown that *Methanopyrus kandleri *most likely groups with *methanococcales *and *methanomicrobiales *(Brochier et al. 2004) and that N. *equitans *is at least sister-group of euryarchaea (if not of Thermococcales). As previously indicated, the grouping of *C. symbiosum *with crenarchaea could be also highly misleading. It should have been interesting to compare the genome content of LACA based on the 16S rRNA phylogeny and the more robust phylogeny based on ribosomal proteins. My feeling is that the nature of LACA (chemo-litoautotroph or not, hyperthermophile or not?) is still a pending question.

**Authors' response: ***We have not really followed the 16S RNA tree but rather deliberately chose a poorly resolved topology so as not to subscribe to any particular phylogenetic hypothesis with respect to issues that are still considered unresolved. We are well aware of the published work on archaeal phylogenies and the two important papers by Brochier et al. are cited. Out of fairness, the likely position of Methanopyrus with Methanococcales and Methanobacteriales, was first reported in Slesarev et al. in 2002, and this cited as well. The wording on Methanopyrus in the text was modified to reflect these reports but we did not modify the tree in *Fig. 7. *One has to keep in mind that the reconstruction here is by no means supposed to be the final word on the scenario of archaeal evolution but more of an exercise showcasing the utility of the arCOGs. We expect that there will be many more iterations with more genomes, better resolved trees, and better methods of reconstruction, and we certainly hope to be involved*.

Finally, in the discussion of the gene-content tree, the authors wrote «*methanogenesis which are spread both vertically and horizontally*». In fact, a detailed phylogenetic analysis of genes involved in methanogenesis by Bapteste and co-workers has shown that, surprisingly, although these proteins can be considered as «operational» they have been only transmitted by vertical inheritance in the archaeal domain (Bapteste et al., 2005).

**Authors' response: ***We believe that the issue is not quite resolved yet. The wording in the paper was softened, nevertheless*.

Bapteste E, Brochier C, Boucher Y.

Higher-level classification of the Archaea: evolution of methanogenesis and methanogens.

Archaea.1, 353–363 (2005).

Brochier, C. Forterre P. and Gribaldo S.

Archaeal phylogeny based on proteins of the transcription and translation machineries: tackling the *Methanopyrus kandleri *paradox

Genome Biology, 5, R17 (2004).

Brochier, C., Gribaldo, S., Zivanovic, Y. Confalonieri, F. and Forterre, P.

Nanoarchaea: representative of a novel archaeal phylum or a fast evolving euryarchaeal lineage related to Thermococcales?

Genome Biology, 6:R42 (2005).

Forterre, P. and Philippe, H

Where is the root of the universal tree of life?

Bioessays, 21, 871–879 (1999).

Lecompte O, Ripp R, Thierry JC, Moras D, Poch O.

Comparative analysis of ribosomal proteins in complete genomes: an example of reductive evolution at the domain scale.

Nucleic Acids Res., 30, 5382–5390 (2002).

### Reviewer 3: Purificación López-García, CNRS, Université Paris-Sud

This article describes the analysis of genes present in most of the currently available archaeal genome sequences in view of their classification in clusters of orthologous genes specific to the archaea (arCOG). It represents an updated extension of previous comparative genomic analyses of COGs though exclusively devoted to the archaea. As a consequence, the arCOG database produced is more refined, resulting in an increased coverage and resolution. The latter is reflected in the numerical increase of specific archaeal COGs and the accompanying decrease in the number of clusters containing paralogs. The comparison of arCOGs thus defined allows to infer the presence of ~166 core arCOGs, which were likely present in the last archaeal common ancestor (LACA), while 282 and 336 arCOGs appear ancestral to the euryarchaeotal and crenarchaeotal branches, respectively. From the nature of the core arCOGs, the authors conclude that the LACA was a rather complex hyperthermophilic chemoautotroph possessing ~1000 genes. Differential gene gain and loss are predicted to have occurred in the two major archaeal branches. The pattern of arCOG distribution in the different archaeal genomes is used to reconstruct a gene-content tree. Despite biases that may be associated to this approach, which are cautiously recognized by the authors, the tree obtained is largely congruent with widely accepted archaeal molecular phylogenies. Interestingly, *Nanoarchaeum equitans *is placed within the Thermococcales in agreement with recent detailed phylogenetic analyses, reinforcing the idea that the basal placement of *N. equitans *in some trees was due to long-branch attraction artifacts. The two major differences of this gene-content tree with respect to previous accepted molecular phylogenies for the archaea are that all methanogenic euryarchaeota, normally split in at least two large groups in molecular phylogenies, cluster together as they share a large number of methanogenesis-related genes, and that *Cenarchaeum symbiosum *is placed within the Euryarchaota, in disagreement with its expected position within the Crenarchaeota. Although the type of analyses carried out is not innovative, the new arCOG database presented here will certainly be very useful to improve future genome annotations.

I have only a few minor comments or suggestions, as follows:

-*First, it has to be noticed that the euryarchaeal core (282 arCOGs) and the crenarchaeal core (336 arCOGs) are not dramatically larger than the pan-archaeal core, emphasizing the general volatility of archaeal genomes*.

The affirmation that 282 and 336 arCOGs *are not dramatically larger *than the 166 core arCOGs appears quite subjective. It is roughly twice the size. How does this compare with the situation in bacteria? It would be nice to include this information here, and even better, to relate/normalize this information to the average genetic distance in a reference conserved genetic marker, such as the 16S rRNA gene.

**Authors' response: ***"Dramatic", certainly, is in the eye of the beholder. We believe the reader will see it that way, so no changes. Comparing to bacteria is dubious because there are no two major groups of bacteria emulating Euryarchaeota and Crenarchaeota. Calibration – complex exercise that goes beyond the scope of this paper*.

Defining genome volatility would also be useful. Genome volatility has been defined in the literature as the mean volatility of all codons weighted by their frequency within the genome, codon volatility being a measurement related to the non-synonymous versus synonymous mutations (e.g. Dagan and Graur, Mol Biol Evol 2004, 22:496). I believe the meaning is more informal and vague here, and also subjective. Can you provide a reference showing that archaeal genomes are "volatile"?

**Authors' response: ***Good point, we changed the wording to avoid any wrong connotations, "volatility" is not used anymore*.

Horizontal gene transfer from bacteria has apparently contributed to shape the *C. symbiosum *genome. In page 14, it is mentioned that *C. symbiosum *falls within the euryarchaeotal part of the gene-content tree. Would you predict that HGT from euryarchaeota may partly explain this observation as some (although very limited) environmental genomic studies appear to suggest (Lopez-Garcia, Brochier et al, Environ Microbiol 2004, 6:19?

**Authors' response: ***Yes, a valid point, we included this possibility in the revision and cite the paper*.

## Authors' contributions

KSM performed the bulk of comparative genome analysis and contributed to the design of computational procedures and algorithms; YIW designed the algorithms and computational procedures, and contributed to genome analysis and software development; AS contributed to software development; EVK initiated the project, contributed to the design of the computational procedure, and wrote the manuscript. All authors read and approved the final version of the manuscript.
